# Molecular analysis of the benthos microbial community in Zavarzin thermal spring (Uzon Caldera, Kamchatka, Russia)

**DOI:** 10.1186/1471-2164-15-S12-S12

**Published:** 2014-12-19

**Authors:** Alexey S Rozanov, Alla V Bryanskaya, Tatiana K Malup, Irina A Meshcheryakova, Elena V Lazareva, Oksana P Taran, Timofey V Ivanisenko, Vladimir A Ivanisenko, Sergey M Zhmodik, Nikolay A Kolchanov, Sergey E Peltek

**Affiliations:** 1 Institute of Cytology & Genetics SB RAS, Novosibirsk, 630090, Russia; 2 V S Sobolev Institute of Geology and Mineralogy SB RAS, Novosibirsk, 630090, Russia; 3 Boreskov Institute of Catalysis SB RAS, Novosibirsk, 630090, Russia; 4 Novosibirsk State University, Novosibirsk, 630090, Russia

**Keywords:** Microbiology, metagenome, Zavarzin thermal spring, Uzon Caldera, Kamchatka

## Abstract

**Background:**

Geothermal areas are of great interest for the study of microbial communities. The results of such investigations can be used in a variety of fields (ecology, microbiology, medicine) to answer fundamental questions, as well as those with practical benefits. Uzon caldera is located in the Uzon-Geyser depression that is situated in the centre of the Karym-Semyachin region of the East Kamchatka graben-synclinorium. The microbial communities of Zavarzin spring are well studied; however, its benthic microbial mat has not been previously described.

**Results:**

Pyrosequencing of the V3 region of the 16S rRNA gene was used to study the benthic microbial community of the Zavarzin thermal spring (Uzon Caldera, Kamchatka). The community is dominated by bacteria (>95% of all sequences), including thermophilic, chemoorganotrophic *Caldiserica *(33.0%) and *Dictyoglomi *(24.8%). The benthic community and the previously examined planktonic community of Zavarzin spring have qualitatively similar, but quantitatively different, compositions.

**Conclusions:**

In this study, we performed a metagenomic analysis of the benthic microbial mat of Zavarzin spring. We compared this benthic community to microbial communities found in the water and of an integral probe consisting of water and bottom sediments. Various phylogenetic groups of microorganisms, including potentially new ones, represent the full-fledged trophic system of Zavarzin. A thorough geochemical study of the spring was performed.

## Background

Microbial communities in geothermal areas are of great interest in a variety of fields, including ecology, microbiology and medicine. They have the potential to answer fundamental questions, as well as those with practical benefits [1-4]. The microbiology of these geothermal habitats has been studied during the last few decades [5,6]. Classical microbiology techniques can only be used to study microorganisms that can be cultured; however, many microbial species, including those from thermal habitats, cannot be cultured in the laboratory. There is a growing realization that uncultured microbiota are untapped resources for basic and applied research. Modern molecular biology techniques have revealed a species richness of microbial systems that has far surpassed that which was expected based on the use of traditional microbiological techniques. The earliest studies of microbial communities in Yellowstone National Park (USA) using analyses of 16S rRNA genes resulted in the detection of many novel, unculturable microorganisms that are active at high temperatures [7,8].

Microbial communities in Kamchatka (Russia) thermal springs have been studied since the 1960s using traditional microbiology methods based on morphology, physiology and biochemistry of the indigenous microorganisms [9].

Subsequent studies of the microbial communities in Kamchatka used more modern molecular biological approaches [10-15] Thus, numerous phylogenetically diverse microorganisms were found in the waters of Zavarzin spring (caldera of the Uzon volcano in the Kronotsky Nature Reserve). Zavarzin is prominent among the Uzon thermal springs. It is a large, deep pool with fine blue sediment and thick layers of sulphur deposits, and microbial communities have developed along its creek [16,17].

Water samples from the Zavarzin thermal spring have only recently been characterized [16]. A metagenomic study of integral water and sediment samples collected from two thermal outlets of the Uzon caldera, Arkashin Shurf and Zavarzin thermal spring, has been performed [17]. The composition of water samples from Zavarzin thermal spring has recently been determined by pyrosequencing [16].

Our aim here was to study the metagenome of the Zavarzin benthic microbial community by 16S rRNA pyrosequencing, and to compare the microbial communities found in the water and the benthos in Zavarzin.

There are two major thermal outlets in Kamchatka, the Uzon caldera and the Geyser Valley. We performed our studies in the Uzon caldera. The Uzon-Geyser depression is located in the centre of the Karym-Semyachin region of the East Kamchatka graben-synclinorium. Uzon caldera is located in the western part of the depression. The caldera of the Uzon volcano is a 150-km^2 ^depression about 10 km in diameter. One of the largest craters made by a volcanic eruption in Kamchatka, which resulted in the formation of Dalneye Lake, is 1.65 km in diameter and is situated in the eastern part of the caldera. This part of the caldera is swampy; several lakes are located there, of these Dalneye is the largest. Multiple springheads of the Shumnaya River flow through the caldera.

Uzon caldera is filled with lake sediments and pumice. Lake sediments are represented by aleuropelitic tuffs and various pumice sands formed at the bottom of volcanic lakes. Dacite extrusions formed at the same tame as lake sediments; the closest extrusion to the Uzon thermal anomaly is Belaya Mountain. Glacial moraines formed during the last glaciation, and peatbog deposits are Holocene deposits. Talus-proluvial gravel-pebble deposits are also found there [18].

**Figure 1:**
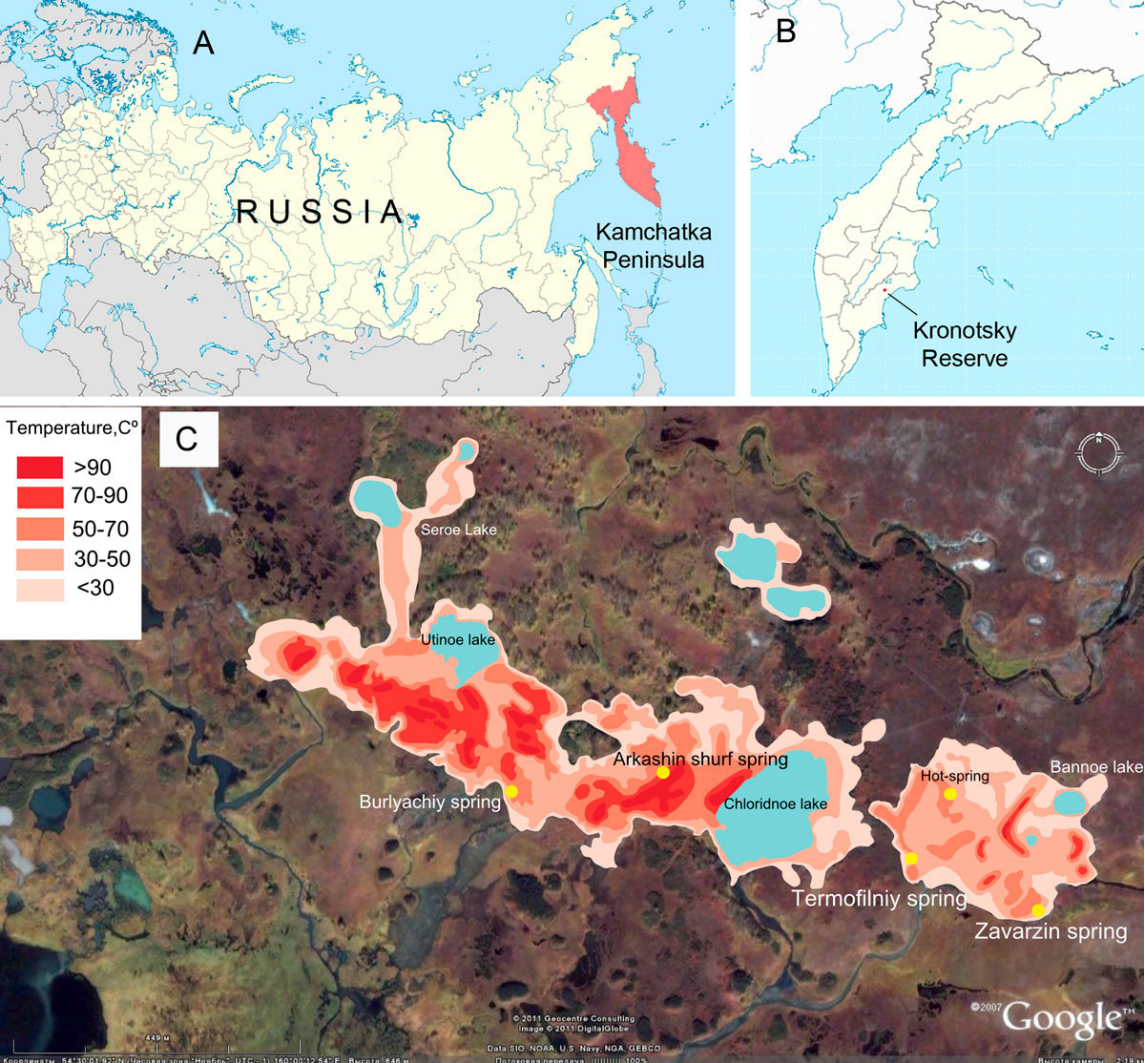
**Location of the Zavarzin thermal spring**. A, B - A map that shows the location of the Kronotsky reserve, Kamchtka Russia. C - A schematic thermometrical map of the East field in the Uzon caldera, Kamchatka, Russia.

In Uzon, recent hydrothermal activity is manifested by outlets of overheated water that produces a zone of local surface warming in its northeastern part. This is the most depressed and swampy part of the caldera. Thermal activity is confined to several regions of various sizes, scattered over an area of about 15 km^2^. There are five thermal fields in the Uzon caldera: South, West, North, Orange and East (Figure 1). The East thermal field is the largest of the five. All types of surface hydrothermal activity are found there: hot springs, water and dirt pools, dirt volcanoes, fumaroles, etc. The field is 1.5 km long, and the heated zone is 200-400 m wide [19]. The field may be divided into three sectors.

All water types characteristic of recent volcanic hydrothermal fields are found in the Uzon caldera. Overheated chloride-sodium solutions are discharged in sector II of the East thermal field (Figure 1), which is characterized by high concentrations of boron, arsenic, antimony, mercury and lithium. The main ore body, consisting mostly of realgar, orpiment, antimonite, pyrite, cinnabar and metacinnabar, is located within sector II [18-20]. The others water types (chloride-sulphate, sulphate-chloride, sulphate and bicarbonate) are believed to be formed by complex processes of the differentiation of highly mineralized chloride-sodium solutions, as well as by its mixing with acid solutions formed by the oxidation of sulphide material and surface hydrocarbonate water. The water of Zavarzin thermal spring located in the extreme south of sector I of the East thermal field, far from the discharge of the main thermal solutions, is precisely the product of such a mixing.

## Results and discussion

### Object of the study

**Figure 2:**
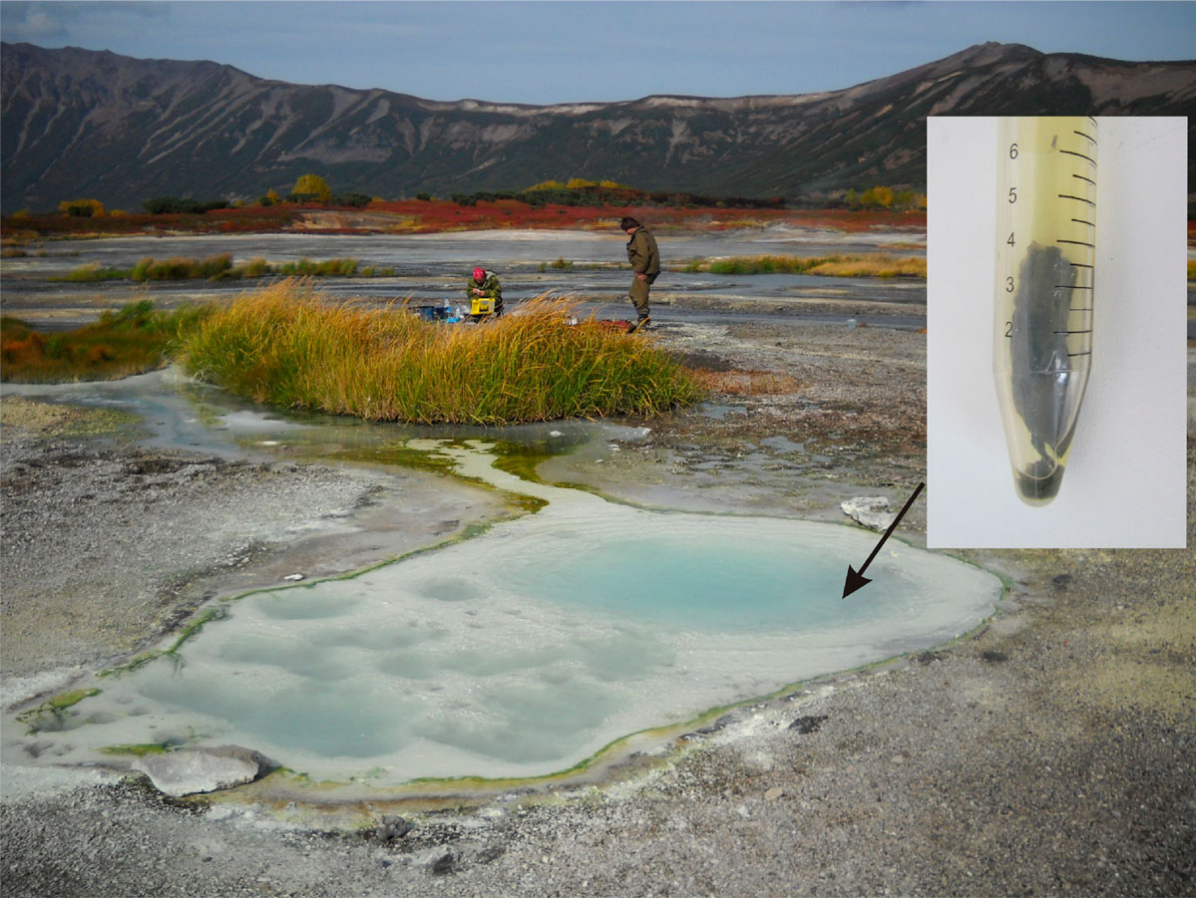
**Zavarzin thermal spring**. Arrow indicates the sampling site.

Zavarzin is a round pool about 3 m in diameter, enclosed in caldera lake sediments. The bottom of the pool is covered with loose, fine, milk-cream-coloured native sulphur bottom sediment. Green cyanobacterial communities are found along the edge of the pool and the course of the stream (Figure 2).

Zavarzin and its microbial communities have been described in detail elsewhere [16,17]. However, the benthic microbial mat, which we found serendipitously in the fall of 2010 when sampling bottom sediments, has not been described.

### Zavarzin water content

The Zavarzin thermal spring water temperature is 56-58°C and the pH is 6.6, which is at the boundary between neutral and acidic water (Table 1). The total dissolved solids (TDS) is 410 mg/l. Its water content is complex: although sodium and hydrocarbonate ions prevail, the content of magnesium, calcium and sulphates is also very high, as is its silica content. As in the Uzon caldera, the water is a sodium chloride hydrothermal solution, with high concentrations of Li (47 ppb), B (980 ppb), Mn (610 ppb), As (22 ppb), Br (47 ppb), I (6 ppb), Cs (2.4 ppb) and Ba (98 ppb). The high contents of Al (33 ppb), P (160 ppb) and Fe (16 ppb,) in the Zavarzin thermal spring water possibly result from a mixed solution formed by the oxidation of sulphides. As in all springs of the Uzon caldera, the concentration of Ag is high (0.8 ppb).

**Table 1:** Zavarzin water content.

Elemental composition	Units	C_w_	Elemental composition	Units	C_w_
Temperature	°C	58	Ga	ppb	0.13

pH	pH	6.62	Ge	ppb	0.7

Eh	mV	54	As	ppb	22

DOC	ppm	1.0	Se	ppb	0.38

Ca^2+^	ppm	18.0	Br	ppb	47

Mg^2+^	ppm	5.6	Rb	ppb	9.5

Na^+^	ppm	35	Sr	ppb	100

K^+^	ppm	4.9	Y	ppb	0.0054

HCO_3_^-^	ppm	180	Zr	ppb	0.011

SO_4_^2-^	ppm	60	Nb	ppb	< 300E-6

H_2_S	ppm	3.6	Mo	ppb	0.022

Cl^-^	ppm	13.5	Ag	ppb	0.04

Si	ppm	85	Cd	ppb	0.003

TDS, calculated	ppm	410	Sn	ppb	0.015

Li	ppb	47	Sb	ppb	0.028

B	ppb	980	Te	ppb	0.006

Be	ppb	0.015	I	ppb	6

Al	ppb	33	Cs	ppb	2.4

P	ppb	160	Ba	ppb	98

Zn	ppb	85	Ta	ppb	< 200E-6

Ti	ppb	0.75	W	ppb	0.083

V	ppb	0.084	Re	ppb	2.00E-04

Cr	ppb	0.11	Hg	ppb	0.8

Mn	ppb	610	Tl	ppb	0.0011

Fe	ppb	16	Pb	ppb	0.13

Co	ppb	0.054	Bi	ppb	0.0015

Ni	ppb	0.17	Th	ppb	< 500E-6

Cu	ppb	0.4	U	ppb	0.0015

### Mineral content of bottom sediments and the microbial community

**Figure 3:**
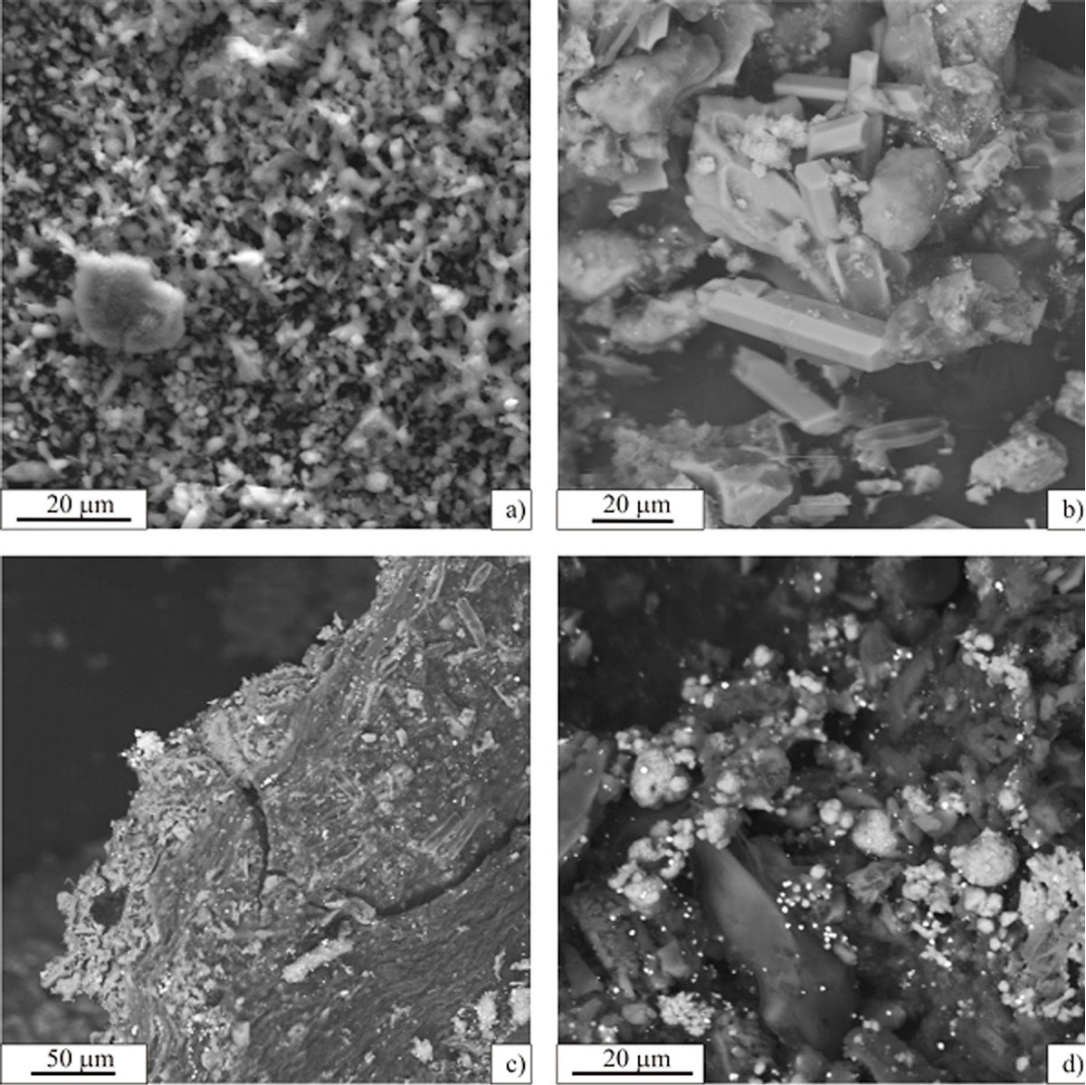
**Mineral deposits of Zavarzin**. (a) drop-like particles of native sulfur, a big opal formation is located in the center. (b) gypsum crystals among igneous rock detritus. (c) organomineral aggregate of the benthos microbial communities includes bacterial filaments, diatom shells, detritus of igneous rock from the Uzon caldera, sphere-like pyrite coalescences regions consolidated by opal, and submicron particles of mercury sulfide. (d) sphere-like pyrite coalescences and submicron particles of cinnabar cover the organomineral aggregate of the benthos community.

As mentioned above, the bottom of Zavarzin is covered with highly dispersed sulphur particles, which have a sphere-like, drop-like or elongated shape (Figure 3a). No faceted crystals are observed. This sulphur layer also includes grains of amorphous silica and the detritus of lake sediments that fill the Uzon caldera. Clusters of well-faceted prismatic crystals of gypsum often occur under the sulphur layer (Figure 3b). The mineral content of the organomineral aggregate of the benthic microbial community is more diverse. A considerable amount of igneous rock detritus is found among bacterial filaments and diatom shells (Figure 3c). In addition, there are local areas of silicification and many sphere-like pyrite coalescences (possibly framboids) up to 10 μm in diameter. Well-faceted, octahedral pyrite crystals (up to 5 μm in diameter) are occasionally found.

### Elemental content of the bottom sediments and microbial community

Table 2 presents the elemental contents of Zavarzin gryphon walls that are represented by fragmented lake deposits (enclosing sediments), highly dispersed sulphur bottom sediments precipitated from water, a benthic microbial community and a cyanobacterial community living along the edge of the gryphon. In comparison to enclosing sediments, highly dispersed sulphur bottom sediments are characterized by much higher contents of Sb and Te, about the same concentrations of Ni, Cu, Br and As, and lower concentrations of other elements; the Hg content is 190 ppm. We used the logarithm of the ratio of the content of an element in the sediments (Cs) to its content in water (Cw) to compare these phases. The log (Cs/Cw) ranged from 1 to 3 for the elements that are mobile in these conditions: alkaline and alkali-earth elements (Na, Ca, Rb, Sr, Cs, K), anionic elements (Ge, Se, V, As), halogenides (Br and I), as well as Mn and Zn. For such elements as Cr, Co, Mo, Ad and Pb, the contents in sediments exceeds that in water by four orders of magnitude, while the contents of Fe, Ni, Cu, Ga, Cd, Sn, Sb, Te, Hg and U were five orders of magnitude greater. Ti, Y, Zr, Nb and Th are the least mobile elements (Table 2). The elemental content of the cyanobacterial community found along the edge of the gryphon is identical to that of sulphur layer.

**Table 2:** Elemental composition in bottom sediments and the benthos microbial community of Zavarzin.

Elemental composition	Units	Sulfur bottom sediments (C_s_)	Cyanobacterial community along the edge of the gryphon	Enclosing lake deposits (C_L_)	Benthos community (C_c_)	C_c_-C_L_	Log (C_s_/C_w_)	Log (C_c_/C_w_)
Ca	%	0.15	0.2	1.7	1.4	~*	2	-

Na	%	0.1	0.08	1.2	1.2	**~**	1	-

K	%	0.25	0.28	1.9	0.85	**~**	3	-

Sc	ppm	1.8	1.4	17.0	17.1	**~**	n.d.	-

Ti	%	0.04	0.03	0.90	0.90	**~**	6	-

V	ppm	< 0.05	< 0.05	160	113	**~**	3	-

Cr	ppm	< 3	< 2	7.5	9	**~**	4	-

Mn	ppm	6	3	89	94	**~**	2	-

Fe	%	0.4	0.3	5.0	4.6	**~**	5	-

Co	ppm	1.3	0.9	13.5	13.3	**~**	4	-

Ni	ppm	10.0	7.5	11.5	12.2	**~**	5	-

Cu	ppm	17.7	19.2	24.5	38.0	13.5	5	5

Zn	ppm	22.5	20.5	66.0	60.5	**~**	2	-

Ga	ppm	5.6	5.0	19.2	16.5	**~**	5	-

Ge	ppm	< 0.05	< 0.05	2.4	< 0.05	**~**	2	-

As	ppm	27	16	52	122	70	3	4

Se	ppm	< 0.05	< 0.05	< 0.05	0.7	0.7	2	3

Br	ppm	7.9	8.7	4.0	82.5	78.5	2	3

Rb	ppm	0.4	0.5	21.5	9.5	**~**	2	-

Sr	ppm	13.5	10.6	280.0	154.0	**~**	2	-

Y	ppm	3.2	2.4	22.6	25.0	**~**	6	-

Zr	ppm	17.0	10.3	141.5	154.5	**~**	6	-

Nb	ppm	0.4	0.5	3.4	3.2	**~**	6	-

Mo	ppm	0.25	0.28	1.40	1.45	**~**	4	-

Ag	ppm	0.42	0.35	0.30	0.30	**~**	4	-

Cd	ppm	0.20	0.40	0.70	0.45	**~**	5	-

Sn	ppm	0.55	1.15	1.50	1.90	**~**	5	-

Sb	ppm	3.4	0.4	1.4	3.0	1.6	5	5

Te	ppm	0.34	< 0.05	< 0.05	0.27	**~**	5	-

I	ppm	0.34	0.32	0.93	0.20	**~**	2	-

Cs	ppm	0.4	0.2	1.3	1.55	**~**	2	-

Hg	ppm	190	163	20	1300	1280	5	6

Pb	ppm	2.7	3.8	10.5	11.7	**~**	4	-

Th	ppm	0.3	n.d.	1.5	1.5	**~**	6	-

U	ppm	0.05	1.4	2.55	0.675	**~**	5	-

The concentration of most elements in the biomineral aggregate of the benthic community is close to that of the sandy material of gryphon walls. By calculating the logarithm of the ratio of the content of an element in the biomineral aggregate (Cc) to that in water (Cw), we can see that the contents of Br and Se in the community aggregate are higher than those in water by three orders of magnitude; by four for As; by five for Cu and Sb; and by six for Hg. This enrichment may be explained by sorption of the elements by the organic substance of the community and their subsequent fixation as sulphide minerals. An excess of sulphide ions may be due to sulphate-reducing bacteria. Additionally, one cannot exclude the specific accumulation of mercury by certain members of the microbial community. In earlier studies [19], the highest Hg content in the Uzon caldera was found in the sediments of Chloridnoye Lake near water gryphons. This implies that the history of its formation was the same as we observed for Zavarzin. We suggest that the mechanism of formation of high Hg content in Chloridnoye Lake is the same.

### Microbial communities

**Figure 4:**
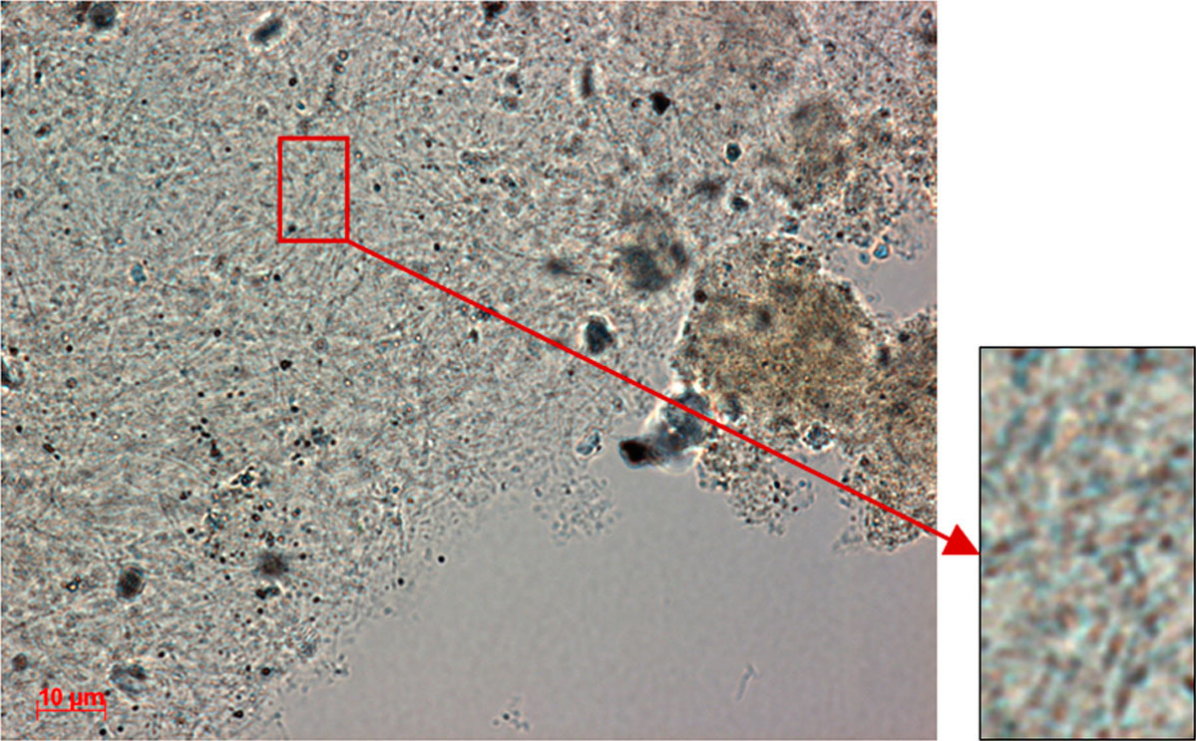
**Dominant morphotypes of the Zavarzin benthos microbial community (location U-1-1)**. Transmitted light microscopy.

**Figure 5:**
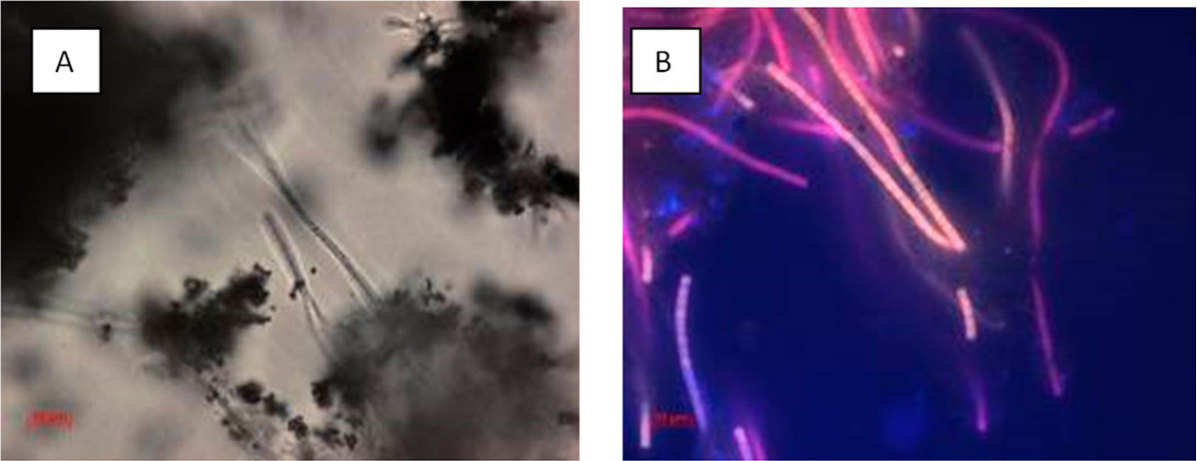
**Morphology of the dominant microorganisms of Zavarzin community (location U-1-2)**. Microscopy A, transmitted light B, fluorescence.

A thin green cyanobacterial film living at 45-49°C is found along the edge of the crater. With the decrease in water temperature along the stream, this cyanobacterial community is replaced gradually by filamentous green algae (Table 3). Further downstream, a thin, green (up to 0.7 cm), easily disintegrated community is present at 35°C (30-40°C). A dark greenish-grey "bushy" community, consisting of thin fields, is found at 22°C. Its appearance persists as temperatures decreased from 22 to 17°C. Colonies of green soil algae develop here, not far from the spring, in water (13°C)-filled depressions made by humans and animals. In the crater itself, under thick layers of sulphur, orange-brown leathery organomineral mat-like structures, about 0.3 cm thick, are found. According to microscopic observations, this mat is formed by filamentous bacteria (Figures 4, 5).

**Table 3:** Types of microbial communities found in Zavarzin

Type of community	Localization site, temperature	Composition of dominant microorganisms
Bottom scale brown organomineral formation	Crater of Zavarzin, depth of 30-50 cm. 56°C	Filamentous bacteria

Thin green flowing single-layer films	Develop along the edge of Zavarzin. About 40°C	Filamentous cyanobacteria

Loose thin dark green-gray epibioses	Develop along the stream branching off from Zavarzin, 20°C	Filamentous cyanobacteria and filamentous green algae

Green epibioses/films on cold soil	Ubiquitous. 10-15°C	Unicellular and colonial green algae

### Composition of the bacterial community

**Figure 6:**
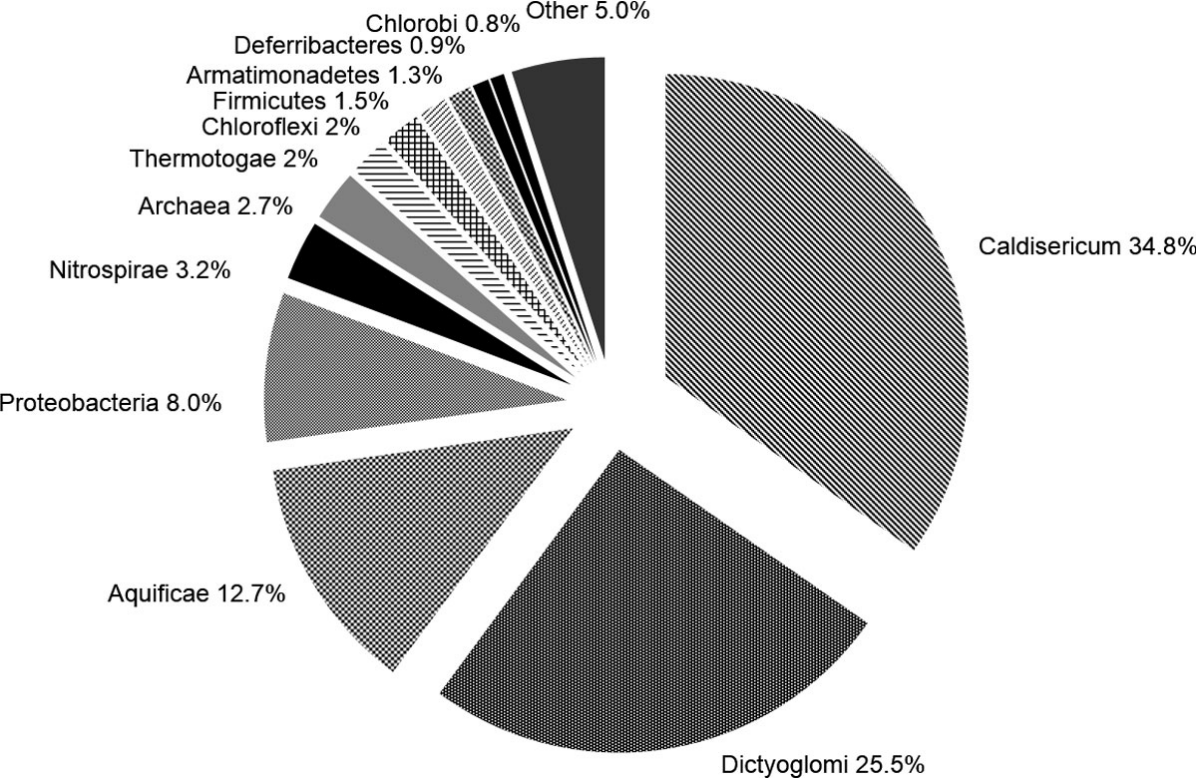
**Dominant microorganisms in the Zavarzin thermal spring benthos community**.

Bacteria of the type *Caldiserica*, which is also widely represented in thermal springs in Japan and Yellowstone (USA), are the most numerous in the studied community (34.8%). The majority of these sequences have 99% sequence similarity to *Caldisericum exile *AZM16c01 (Figure 6). About 1.0% of sequences are not closely related to any cultured microorganisms. The representatives of this type are filamentous thermophiles and heterotrophs, with growth optima ranging from 60-75°C, and pH optima ranging from 5.5-7.5 [21]. About 0.4% of sequences of this type are 98% similar to bacteria from hot springs in Papua New Guinea [22] and Yellowstone [23]. Most represented species of prokaryotes are show in Table 4

**Table 4:** Microbial community of the Zavarzin benthos mat.

Domain	Phylum	% of the total sequence set	Most closely related 16S sequence	Sequence similarity (%)
Bacteria	Caldiserica	33.4	*Caldisericum exile *AZM16c01	98

		1.0	Uncultured bacterium clone ZB_P11_J22	99

		0.4	Uncultured bacterium clone OPB14	98

	Dictyoglomi	25.5	*Dictyoglomus turgidum *DSM 6724	99

	Aquificae	11.3	*Sulfurihydrogenibium yellowstonense *strain SS5	99

		1.3	*Thermosulfidibacter takaii *strain ABI70S6	98

		0.8	Uncultured *Aquificales *bacterium clone YNP_SBC_QL1_B3	89

	Deltaproteobacteria*	6.0	*Desulfurella kamchatkensis *K-119	100

		1.3	*Syntrophorhabdus aromaticivorans *UI	96

	Nitrospirae	1.2	*Thermodesulfovibrio yellowstonii *DSM 11347	100

		2.0	Uncultured *Nitrospirae *bacterium clone OTU74	96

	Thermotogae	2.0	*Fervidobacterium nodosum *Rt17-B1	99

	Bacteroidetes/ Chlorobi	0.1	*Chitinophagaceae *bacterium enrichment culture clone NB-10	97

		0.4	*Chlorobi *bacterium enrichment culture clone phylotype P1	97

		0.7	Uncultured *Chlorobium *sp. clone OPPB063	94

	Thermodesulfobacteria	1.2	*Caldimicrobium rimae *strain DS 16S	99

	Candidate divisions	1.1	Candidate division clone	-

	Other	6.0		

Archaea	Crenarchaeota	0.4	Uncultured Crenarchaeota 16S rRNA gene, clone C10	95

		0.3	Uncultured crenarchaeote clone A14-32	95

		0.2	Uncultured crenarchaeote clone A14-45	96

	Thaumarchaeota	1.6	Uncultured thaumarchaeote clone 8_Kam37	96

	Euryarchaeota	0.1	Uncultured archaeon clone ZA_P5_B12	97

		0.3	Uncultured archaeon 16S rRNA gene, clone N8-H5	93

The next most abundant group contains sequences related to the *Dictyoglomi *type (25.5%) These microorganisms are abundant in thermal habitats [24]. Almost all sequences are very similar or identical to the *Dictyoglomus turgidum *DSM 6724 strain, which was isolated from Kamchatka thermal springs [25]. These bacteria are filamentous obligate anaerobes and chemoorganotrophs; their optimal temperature is about 70°C and their pH optima range from 6.0-9.0 [26].

Bacteria of the *Aquificae *type are usually dominant in thermal waters with neutral pH, and 12.7% of the sequences fall into this type. Members of the *Aquificae *are heterotrophs or chemolythotrophs, microaerophiles or obligate anaerobes [27]. About 11.3% of the sequences we analysed belong to the *Sulfurihydrogenibium *genus. One of the species of this genus, *Sulfurihydrogenibium rodmanii *is found in the Kamchatka hydrotherms [28]. Our sequences show 99% sequence similarity to *Sulfurihydrogenibium yellowstonense *SS5 [29]. This species is a chemolithoautotroph and can oxidize sulphur in microaerophilic conditions. Another 1.3% of sequences belong to members of the *Thermosulfidibacter *genus, and have 98% sequence similarity to *Thermosulfidibacter takaii *ABI70S6, which is an obligate anaerobe, a hydrogen-oxidizing chemolithoautotroph and a sulphate reducer, with growth optimum at 70°C and pH optima ranging from 5.0-7.5 [30].

The *Proteobacteria *type in the Zavarzin community is represented by the *Deltaproteobacteria*, Betaproteobacteria and Gammaproteobacteria classes. 6.9% of the sequences are identical to Desulfurella kamchatkensis K-119 (*Deltaproteobacteria*), which was isolated from the Kamchatka thermal springs. Representatives of this genus are obligate anaerobes, sulphate reducers, can live on media containing hydrogen and sulphur, and may act as heterotrophs, processing organic acids to CO^2 ^and water. These bacteria have growth optima ranging from 50-70°C and pH optima ranging from 6.0-8.0 [31]. The remaining 0.6% have 96% sequence similarity to *Syntrophorabdus aromaticivorans *UI, which is an obligate anaerobe, can oxidize phenol, and has a growth optimum at 37°C and pH optima ranging from 6.0-7.0 [32].

Sequences belonging to the *Nitrospirae *type constitute 3.3% of all sequences; 1.2% are identical to *Thermodesulfovibrio yellowstonii *DSM 11347 [33]. The remaining 2.0% are 97% similar to a bacterium in thermal springs in Thailand [34], which is placed within the *Nitrospirae*, despite its low sequence similarity (78%) to other representatives of this type [35]. The sequence is 89% similar to an uncultured *Dehalococcoides *(Chloroflexi) [36] and 89% similar to other published sequences [37]. Therefore, its phylogenetic position requires further revision.

The *Thermotogae *type is represented by 2.0% of sequences, all of which are 99% similar to *Fervidobacterium nodosum *Rt17-B1, extracted from thermal springs in New Zealand [38,39]. This microorganism is an obligate anaerobe and a chemoheterotroph, which actively grows at 50-80°C and pH 6.0-8.0.

Approximately 1.5% of the sequences belong to the *Firmicutes *type and have 93% to 98% similarity to representatives of the *Clostridia *class (1.4%). Half are 94% similar to *Clostridium cellulosi*, which is an obligate anaerobe and a heterotroph, with a growth optimum at neutral pH and 55-60°C [40]. About 0.1% of the sequences have 93% sequence similarity to *Eubacterium *sp. from Yellowstone cyanobacterial mats [41]. Another 0.1% is 98% similar to *Clostridium symbiosum*, and the remaining 95% are similar to *Desulfotomaculum geothermicum *DSM 3669, which is an obligate anaerobe, a heterotroph and a hydrogen-oxidizing, sulphate reducer [42].

About 1.2% of all sequences belong to the *Thermodesulfobacteria *type. All are 99% similar to *Caldimicrobium rimae *DS, an obligate anaerobe, a facultative autolithotroph, which can grow in the presence of hydrogen and thiosulfate, using CO^2 ^as a carbon source; it's growth optimum is at pH 6.8-7.5 and 52-82°C [43].

Another 0.1% of sequences have sequence similarity to bacteria of the *Actinobacteria *type. They have 94% sequence similarity to uncultured, rhizospheric *Gaiella occulta *F2-233 [44] and are not closely related to any cultured thermophilic microorganism.

About 1.4% of our sequences had 93-100% sequence similarity to the following candidate types: AC1, OP1 and OD1. Our sequences falling into OP1 are closely related to those from Uzon [17] and Central Tibet [45]. Representatives of the *Armatimonadetes *(1.3%) are closely related (99%) to the uncultured bacteria found in Uzon [17], Yellowstone [8] and Central Tibet [45].

Approximately 2.6% of our sequences are not closely related to culturable microorganisms, but are >99% similar to the sequences obtained from environmental samples from the Uzon caldera [17].

Representatives of the *Deferribacteres *type make up 0.8% of our sequences. They are 99% similar to *Calditerrivibrio nitroreducens *DSM 19672 from thermal springs in Japan, which is an obligate anaerobe, a heterotrophic nitrate reducer that reduces nitrate to ammonium, and which has a growth optimum of 55°C and pH 7.0-7.5 [46].

*Chloroflexi *accounted for less than 2% of all sequences. Half of these were highly similar to *Chloroflexus aurantiacus *J-10-fl from Japanese hot springs, a photosynthetic autotroph that can also grow heterotrophically in darkness at 52-60°C and pH 8.0.

### Archaeal community

About 2.7% of the obtained sequences fall into the Archaea domain. Representatives of *Crenarchaeota *(37%), *Euryarchaeota *(3.7%), and *Thaumarchaeota *(59%) were found. *Euryarchaeota*-related sequences are 97% similar to those of the uncultured microorganisms obtained from the Uzon caldera [47]. Sequences belonging to the *Crenarchaeota *were closely related to those from the Uzon caldera obtained by other authors, and less similar (95%) to those from thermal springs in Bulgaria [48], Mexico [49] and China [50]. *Thaumarchaeota*-like sequences were 96% similar to the sequences of the archaea found in the Kamchatka thermal springs [51,52].

### Metabolism of the benthic microbial community

In this study, we obtained information on the structure and composition of the benthic microbial community in the Zavarzin spring. Given the high degree of sequence similarity that was characteristic of most obtained sequences, assumptions regarding the metabolic pathways characteristic of the community and its interactions with the water microbial community appear reasonable.

Gumerov et al. [16] have suggested that the primary production of organic substances in Zavarzin may be formed both by photosynthesis and chemolithoautotrophy. *Chloroflexi *(2.0%) and chemolithotrophs (about 15%) account for significantly smaller numbers of sequences in comparison with the water and cyanobacterial mats of Zavarzin. It was demonstrated that chemolithooautotrophic production may be performed by the following groups: the *Aquificae *(*Sulfurihydrogenibium yellowstonense *SS5, 11.3%, and *Thermosulfidibacter takaii*, 1.3%) and the *Thermodesulfobacteria *(*Caldimicrobium rimae *DS, 1.2%) [33,34,49]. It is evident that the main production of organic substances in Zavarzin takes place in the water and the cyanobacterial mat. However, Burgess et al. [17], who searched for primary producers in an integral sample from Zavarzin, did not find any primary producers, suggesting that this ecosystem depends on an allochthonous carbon source.

Most sequences that we obtained from the benthic mat belong to decomposers of organic substances. These are organotrophs that form the structure of the community. Over 50% of the sequences belong to the *Caldiserica *(34.8%) and *Dictyoglomi *(25.5%), while *Proteobacteria*, *Thermotogae*, *Firmicutes *and *Deferribacteres *are less abundant. Decomposition of organic substances may be achieved either by fermentation or by complete oxidation using oxygen, nitrate, sulphur or thiosulfate as electron acceptors. Most microorganisms from the benthic mat use sulphur, which is formed by the oxidation of hydrogen sulphide in the water, as an electron acceptor. No organisms capable of nitrification were found in the water of Zavarzin. However, they were found in the benthic mat: *Archaea *belonging to the *Thaumarchaeota *type (1.6%) and bacteria belonging to the *Nitrospirae *(3.3%).

Thus, the decomposition of the organic substances that are produced in the water in the cyanobacterial mat is the major process that takes place in the benthic microbial mat.

### A comparison of the microbial communities in water, benthos, and the integral sample

In their work [17] concerned with thermal ecosystems of the Uzon caldera, Burgess et al [17] used cloning to determine the microbial composition in a Zavarzin thermal spring integral sample. Two previous studies [16,17], and our current study, are based on pyrosequencing, allowing direct comparisons of microbial communities in the water and the benthic mat of the Zavarzin thermal spring.

Microbial communities of the benthos and water of Zavarzin are similar in composition, but have considerable quantitative differences, as the dominant microorganisms clearly demonstrate (Table 5), that is, the representatives of the *Caldiserica *and *Dictyoglomi*, which are able to form a hard matrix and the structure of the benthic mat, are only weakly represented in the water. Representatives of the *Aquificae*, which are dominant in surface hydrothermals at hyperthermophilic temperatures, are prevalent in the water, but less abundant in the benthic mat.

**Table 5:** Composition of the microbial communities in water, integral sample, and benthos mat in Zavarzin thermal spring.

Domain	Phylum	% in benthos mat	% in water sample	% in integral sample **
Bacteria	Caldiserica	34.8	1.72	1

	Dictyoglomi	25.5	1.65	2

	Aquificae	12.7	32.27	3

	Proteobacteria	8.0	23	13

	Alphaproteobacteria*	-	> 1	-

	Deltaproteobacteria*	7.3	3.01	-

	Betaproteobacteria*	0.3	6.57	13

	Gammaproteobacteria*	0.4	10.99	-

	Epsilonproteobacteria*	-	> 1	-

	Nitrospirae	3.2	-	3

	Thermotogae	2.0	6.27	-

	Firmicutes	1.5	> 1	0.3

	Thermodesulfobacteria	1.2	6.32	-

	Bacteroidetes/Chlorobi	0.8	> 1	-

	Actinobacteria	0.1	> 1	-

	Chloroflexi	2.0	> 1	38

	Deferribacteres	0.9	12.15	8

	Armatimonadetes	1.3	-	6

	Candidate divisions AC1, OP1, OD1	1.4	-	-

	Acidobacteria	0.3	> 1	1

	Deinococcus-Thermus	-	4.35	-

	Verrucomicrobia	-	1.01	-

	Candidate division BRC1	0.1	1.70	0.3

	Cyanobacteria	-	> 0.20	-

	New lineage 1	-	1.04	+

	New lineage 2	-	-	-

	Unclassified	3.8	-	24

Archaea	Crenarchaeota	1.0	2.25	59

	Thaumarchaeota	1.6	-	-

	Euryarchaeota	0.1	1.89	7

	Korarchaeota	-	0.34	21

	Nanoarchaeota	-	0.02	-

	Unclassified	-	-	13

*class

** % of bacterial (300) and archaeal (91) sequences

+ this group was found in the integral sample, but the percentage of clones was not specified

Other microorganisms that are dominant in the water sample are either absent or scarce in the benthic mat. The *Proteobacteria *type is widely represented in the water (*Alpha*-, *Beta*-, *Gamma*- and *Epsilonproteobacteria*), while only *Deltaproteobacteria *are abundant in the benthic mat.

*Thermotogae*, *Thermodesulfobacteria *and *Deferribacteres *are also less abundant in the benthic mat. No representatives of Deinococcus-Thermus, *Verrucomicrobia *and *Cyanobacteria*, as well as no archaea belonging to the Korarchaeota and Nanoarchaeota, are found in the benthos mat.

Candidate types represent 0.3% to 6% of this community, but only one type (BRC1) was found in the water; this type belongs to soil and rhizosphere microorganisms that were probably transferred from nearby locations to the soil of Zavarzin. In addition, no representatives of *Nitrospirae *and *Thaumarchaeota *are found in the water.

A phylogenetic clade endemic to Zavarzin was detected in the water and in the integral sample. This clade is present in the benthic mat as well: sequences that have no sequence similarity to cultured microorganisms, but are closely related (>99%) to the sequences from the study of Burgess et al [17].

## Conclusions

Pyrosequencing of the benthic mat of Zavarzin allowed us to study the composition and structure of microbial communities of Zavarzin, the distribution of some of its components, as well as some of their functional characteristics. The Zavarzin community is a full-fledged trophic system containing various phylogenetic groups of microorganisms, including some potentially new ones. Geochemistry dramatically influences the structure and metabolism of microbial communities, as both are directly associated with the environment and depend on strict conditions. Therefore, in contrast to the water of the spring, only sessile microorganisms with particular metabolism types, adapted to strict anaerobic conditions and able to live on certain substrates can inhabit the benthic microbial mat of Zavarzin.

Geothermal systems that exist in areas of recent volcanism are characterized by common geochemical parameters, e.g., high temperature, lack of oxygen, presence of sulphides and many rare and trace metals, etc. However, despite extreme conditions, these ecosystems harbour a great diversity of microorganisms [53]. The fact that DNA sequences belonging to microorganisms of various taxa, including new phylogenetic groups, have been found in Zavarzin, and other extreme ecosystems as well, indicates that the microbial population of our planet is insufficiently studied, and suggests that extensive speciation may take place in areas of recent volcanism.

It is noteworthy that some elements accumulated in this benthic community, such as Br and Se, are enriched by three orders of magnitude in comparison with the water of this spring, while others, such As, Cu and Sb, and Hg, are enriched by four, five, and six orders of magnitude, respectively. These elements may be adsorbed either actively or passively with subsequent fixation as sulphide minerals. This fact may dramatically influence the composition and structure of the community, and may also enhance the potential mutagenic effects of some of these elements.

A multitude of new microorganisms, including those having unique metabolic pathways, have recently been described, and many biotechnologically important genes and enzymes have been cloned [54]. With this in mind, it is hoped that this ecosystem will continue to give insights into microbial biodiversity. However, studying these ecosystems remains important for biodiversity, conservation and biotechnology. We are planning further studies of various hydrothermal outlets in Kamchatka.

## Methods

### Sampling and microscopy

Mat pieces were collected using a sterile sampler and placed into sterile containers and stored at 4°C. Some samples were fixed using 4% formalin or 50% ethanol. Mat samples were studied using light and fluorescence microscopy (Axioskop A1; Axioimager Z1, Carl Zeiss, Germany) in the Microscopy Centre (IC&G SB RAS, Novosibirsk).

### Chemical analysis of water and the benthic mat

Unstable water parameters were analysed in situ: O_2 _content (commercial test, Merck, Germany) and pH and Eh ("Anion 4100" calibrated using standard solutions). The basic absolute error of pH measurement was ±0.1. Temperature was determined using an electronic thermometer. Water was filtered through a 0.45-μm membrane filter, and 2 ml of distilled HNO_3 _were added per 0.5 l of water for metal content analysis. Samples for basic ion content measurements were kept without preservation.

Water composition and trace element concentrations were determined using a set of methods that enabled cross-checking of the results. Atomic emission spectrometry with inductively coupled plasma (ICP-AS) (Optima 4300 DV), capillary electrophoresis, ICP-MS, and atomic absorption methods were used. Inorganic and organic carbon in solutions were measured using a Total Organic Carbon Analyzer, TOC-VCSH (Shumadzu, Japan). Mercury content was determined using atomic absorption spectrometry by the cold steam method (analyst Zh.O. Badmaeva).

The samples of the microbial community and bottom sediments were analysed via the SR-XRF method (K, Ca, Ti, V, Cr, Mn, Fe, Ni, Cu, Zn, Ga, Ge, Se, Br, Rb, Sr, Y, Zr, Nb, Mo, Ag, Cd, Sn, Sb, Te, Cs, As, Pb, Th and U).

The SR-XRF method was used at the element analysis station VEPP-3 at the synchrotron emission Siberian Centre of the Nuclear Physics Institute SB RAS. An energy-dispersive X-ray optical SR-XRF scheme was applied in two modes of the primary monochromatic emission, 23 and 36 KeV [55]. Processing of the spectra was conducted using the program AXIL. The contents of the elements were calculated using standard samples: BIL-1 (Baikal silt), 1633A (fly ash, National Bureau of Standards, USA), soil-7 (IAEA standard). For determining some of the elements, the instrumental neutron activation method was used.

The LEO 1430VP scanning electron microscope was used to examine the micromorphology and to determine the contents of minerals formed in the microbial communities.

### PCR amplification of metagenomic DNA

Metagenomic DNA extracted from the Zavarzin benthic microbial community was used as a template for amplification of bacterial 16S rRNA genes with universal primers: U341F (5'-CCTACGGGRSGCAGCAG-3', where R is A or G, S is G or C) and U515R (5'-TTTCCGCGGCKGCTGVCAC-3', where K is G or R, R is A or G, V is A, G, or C). Reagents for PCR (DMSO, PCR buffer, polymerase, nucleotide triphosphates) were products of Agilent Technologies, USA. 50 μl of PCR mix contained 1× Herculase buffer, 10 μM of each dNTPs, 10 pmoles of forward and reverse primers, 100 ng of DNA, and 2.5 u of Herculase. The following amplification profile was used: 3 min at 95°C; 6 cycles of 15 s at 95°C, 15 s at 50°C, and 30 s at 72°C; 35 cycles of 10 s at 95°C, 10 s at 55°C, and 30 s at 72°C; and an additional elongation phase of 5 min at 72°C. Amplified products were purified using commercial kits (Fermentas, Lithuania) and used for an additional PCR reaction with primers containing marker sequences that were designed for the "One-Way Reads" sequencing protocol, according to the manufacturer's instructions (Roche, Switzerland). Reamplification was performed using the same profile as for the initial amplification. The obtained PCR fragments were purified by electrophoresis in 1% agarose gels.

### Pyrosequencing

Pyrosequencing of the variable V3 region of the 16S rRNA gene was performed on a GS Junior System sequencer (Roche) using the "ONE-WAY READS AMPLICON SEQUENCING" protocol at the Engelhardt Institute of Molecular Biology RAS by Dr. A.V. Kudriavtseva.

### Sequence analysis

A preliminary, quality filtering of 847 short nucleotide sequences (reads) was done with the PRINSEQ tool [56]. Then, 16S rRNA gene sequence reads with a mean length of about 187 bp were filtered, de-noised and processed by the QIIME software package [57] using the USEARCH sequence analysis tool [58] implemented into the QIIME. The pipeline, including 11 steps, was performed. In the first step, sequences were sorted by length. In the second step, sequences were de-replicated, and the output file had unique sequences only, where each sequence description contained information regarding how many sequences exactly matched the sequence in question. In the third step, de-replicated sequences were sorted by abundance using the information from step 2. In the forth step, sequences were clustered at 97% identity. In the fifth step, de-novo chimera checking using UCHIME [59] was performed. In the sixth step, reference-based chimera checking against the Gold database (http://drive5.com/uchime/gold.fa) was made. In the next two steps, sequences tagged as non-chimeric during steps 6 and 7 were combined and sorted by abundance, and clusters with less than four reads were discarded. In the last three steps, an operational taxonomic units (OTU) picking, with an identity level greater than 0.80, was performed; each non-chimeric read was assigned to the specific OTU identifier to which it belongs. In summation, 834 reads were clustered into 144 OTUs. The representative sequence for each OTU was queried against the GREENGENES database [60] with a make_otu_table.py script from QIIME.

## Competing interests

The authors declare no potential conflict of interest with respect to financial or non-financial competing interests, the authorship and/or publication of this article.

## Authors' contributions

AVB, EVL, OPT and SMZ collected the samples. AVB performed the microscopy. EVL and OPT performed chemical and hydrogeological analyses. TKM and ASR extracted the DNA and processed samples for sequencing, TVI and VAI performed computational and statistical analyses. ASR and IAM planned the sequencing experiment. ASR, AVB and SEP wrote the manuscript, and all authors edited and commented on the paper. SME and NAK planned the expedition.

## Declarations

Publication of this article has been funded by the Russian Science Foundation grant No 14-24-00123.

This article has been published as part of *BMC Genomics *Volume 15 Supplement 12, 2014: Selected articles from the IX International Conference on the Bioinformatics of Genome Regulation and Structure\Systems Biology (BGRS\SB-2014): Genomics. The full contents of the supplement are available online at http://www.biomedcentral.com/bmcgenomics/supplements/15/S12.

## References

[B1] Huber R Huber H Stetter K Towards the ecology of hyperthermophiles: Biotopes, new isolation strategies and novel metabolic properties FEMS Microbiol Rev 2000 24 615 623 10.1111/j.1574-6976.2000.tb00562.x 11077154

[B2] Robb F Antranikian G Grogan D Driessen A Thermophiles: biology and technology at high temperatures 2008 Boca Raton: CRC Press

[B3] Burra PV Kalmar L Tompa P Reduction in Structural Disorder and Functional Complexity in the Thermal Adaptation of Prokaryotes PLoS ONE 2010 5 e12069 10.1371/journal.pone.0012069 20711457 PMC2920320

[B4] Youssef NH Couger MB Elshahed MS Fine-Scale Bacterial Beta Diversity within a Complex Ecosystem (Zodletone Spring, OK, USA): The Role of the Rare Biosphere PLoS ONE 2010 5 e12414 10.1371/journal.pone.0012414 20865128 PMC2932559

[B5] Brock TD Thermophilic microorganisms and life at high temperatures 1978 New York: Springer-Verlag

[B6] Bonch-Osmolovskaia EA Studies of thermophilic microorganisms at the Institute of Microbiology, Russian Academy of Sciences Mikrobiologiia 2004 73 644 658 15595517

[B7] Barns SM Fundyga RE Jeffries MW Pace NR Remarkable archaeal diversity detected in a Yellowstone National Park hot spring environment Proc Natl Acad Sci USA 1994 91 1609 1613 10.1073/pnas.91.5.1609 7510403 PMC43212

[B8] Hugenholtz P Pitulle C Hershberger KL Pace NR Novel division level bacterial diversity in a Yellowstone hot spring J Bacteriol 1998 180 366 376 9440526 10.1128/jb.180.2.366-376.1998 PMC106892

[B9] Kuznetsov SI Microorganisms of hot springs of Kamchatka Tr Latv Padomju Soc Repub Zinat Akad Mikrobiol Inst 1995 4 130 154 13312082

[B10] Zavarzin GA Microbial diversity studies at the Winogradsky Institute of Microbiology Mikrobiologiia 2004 73 598 612 15595513

[B11] Slepova TV Sokolova TG Lysenko AM Tourova TP Kolganova TV Kamzolkina OV Karpov GA Bonch-Osmolovskaya EA Carboxydocella sporoproducens sp. nov., a novel anaerobic CO-utilizing/H2-producing thermophilic bacterium from a Kamchatka hot spring Int J Syst Evol Microbiol 2006 56 797 800 10.1099/ijs.0.63961-0 16585697

[B12] Galchenko VF Barinova ES Podosokorskaya OA Akimov VN Bacterial communities of Uzon caldera hydrotherms (Kamchatka) International Conference «Microorganisms and biosphere», november 19th - 20th, Moskow 25 26

[B13] Chernyh NA Perevalova AA Slepova TV Slobodkina GB Bonch-Osmolovskaia EA Phylogenetic analysis of natural microbial communities and enrichment culturesfrom hydrotherms of Uzon caldera International Conference «Microorganisms and biosphere», november 19th - 20th, Moskow 2007 145 146

[B14] Kublanov IV Perevalova AA Slobodkina GB Lebedinsky AV Bidzhieva SK Kolganova TV Kaliberda EN Rumsh LD Haertlé T Bonch-Osmolovskaya EA Biodiversity of Thermophilic Prokaryoteswith Hydrolytic Activities in Hot Springs of Uzon Caldera, Kamchatka (Russia) Appl Environ Microbiol 2009 75 286 10.1128/AEM.00607-08 18978089 PMC2612224

[B15] Mardanov AV Gumerov VM Beletsky AV Perevalova AA Karpov GA Bonch-Osmolovskaya EA Ravin NV Uncultured archaea dominate in the thermal groundwater of Uzon Caldera, Kamchatka Extremophiles 2011 15 365 372 10.1007/s00792-011-0368-1 21512891

[B16] Gumerov VM Mardanov AV Beletsky AV Bonch-Osmolovskaia EA Ravin NV Molecular analysis of microbial diversity in the Zavarzin Spring, the Uzon caldera Mikrobiologia 2011 80 258 265 21774190

[B17] Burgess EA Unrine JM Mills GL Romanek CS Wiegel J Comparative geochemical and microbiological characterization of two thermal pools in the Uzon Caldera, Kamchatka, Russia Microb Ecol 2012 63 471 489 10.1007/s00248-011-9979-4 22124570

[B18] Byhckov AYu A geochemical model of current ore formation in Uzon caldera (Kamchatka). Moskow: GEOS 2009

[B19] Naboko SI Volcanism,hydrothermal processes, and ore formation. Moskow: Nedra 1974

[B20] Migdisov AA Bychkov AY The behaviour of metals and sulphur during the formation of hydrothermal mercury-antimony-arsenic mineralization, Uzon caldera, Kamchatka, Russia J Volcanol Geotherm Res 1998 84 153 171 10.1016/S0377-0273(98)00038-9

[B21] Mori K Yamaguchi K Sakiyama Y Urabe T Suzuki K Caldisericum exile gen. nov., sp. nov., an anaerobic, thermophilic, filamentous bacterium of a novel bacterial phylum, Caldiserica phyl. nov., originally called the candidate phylum OP5, and description of Caldisericaceae fam. nov., Caldisericales ord. nov. and Caldisericia classis nov Int J Syst Evol Microbiol 2009 59 2894 2898 10.1099/ijs.0.010033-0 19628600

[B22] Amend J-P Burcea L-C Meyer-Dombard D-R Microbial diversity in alkaline hot springs of Ambitle Island, Papua New Guinea GenBank Database 2011 http://www.ncbi.nlm.nih.gov/nuccore/JF935228.1 10.1007/s00792-014-0657-6 24903703

[B23] Meyer-Dombard DR Swingley W Raymond J Havig J Shock EL Summons RE Hydrothermal ecotones and streamer biofilm communities in the Lower Geyser Basin, Yellowstone National Park Environ Microbiol 2011 13 2216 2231 10.1111/j.1462-2920.2011.02476.x 21453405

[B24] Saiki T Kobayashi Y Kawagoe K Beppu T Dictyoglomus thermophilum gen. nov., sp. nov., a Chemoorganotrophic, Anaerobic, Thermophilic Bacterium Int J Syst Bacteriol 1985 35 253 259 10.1099/00207713-35-3-253

[B25] Svetlichny VA Svetlichnya TP Dictyoglomus turgidus, sp. nov., a new extreme thermophilic eubacterium isolated from hot springs in the Uzon Volcano crater Mikrobiologiia 1988 57 435 441

[B26] Krieg NR Staley JT Brown DR Hedlund BP Paster JB Ward NL Ludwig W Whitman WB Bergey's Manual of Systematic Bacteriology 2010 New York: Springer

[B27] Spear JR Walker JJ McCollom TM Pace NR Hydrogen and bioenergetics in the Yellowstone geothermal ecosystem Proc Natl Acad Sci USA 2005 102 2555 2560 10.1073/pnas.0409574102 15671178 PMC548998

[B28] O'Neill AH Liu Y Ferrera I Beveridge TJ Reysenbach AL Sulfurihydrogenibium rodmanii sp. nov., a Sulfur Oxidizing Chemolithoautotroph from the Uzon Caldera, Kamchatka Peninsula, Russia, and Emended Description of the Genus Sulfurihydrogenibium Int J Syst Evol Microbiol 2008 58 1147 1152 10.1099/ijs.0.65431-0 18450704

[B29] Nakagawa S Shtaih Z Banta A Beveridge TJ Sako Y Reysenbach AL Sulfurihydrogenibium yellowstonense sp. nov., an extremely thermophilic, facultatively heterotrophic, sulfur-oxidizing bacterium from Yellowstone National Park, and emended descriptions of the genus Sulfurihydrogenibium, Sulfurihydrogenibium subterraneum and Sulfurihydrogenibium azorense Int J Syst Evol Microbiol 2005 55 2263 2268 10.1099/ijs.0.63708-0 16280480

[B30] Nunoura T Oida H Miyazaki M Suzuki Y Thermosulfidibacter takaii gen. nov., sp. nov., a thermophilic, hydrogen-oxidizing, sulfur-reducing chemolithoautotroph isolated from a deep-sea hydrothermal field in the Southern Okinawa Trough Int J Syst Evol Microbiol 2008 58 659 665 10.1099/ijs.0.65349-0 18319474

[B31] Miroshnichenko ML Rainey FA Hippe H Chernyh NA Kostrikina NA Bonch-Osmolovskaya EA Desulfurella kamchatkensis sp. nov. and Desulfurella propionica sp. nov., new sulfur-respiring thermophilic bacteria from Kamchatka thermal environments Int J Syst Bacteriol 1998 48 475 479 10.1099/00207713-48-2-475 9731287

[B32] Qiu YL Hanada S Ohashi A Harada H Kamagata Y Sekiguchi Y Syntrophorhabdus aromaticivorans gen. nov., sp. nov., the first cultured anaerobe capable of degrading phenol to acetate in obligate syntrophic associations with a hydrogenotrophic methanogen Appl Environ Microbiol 2008 74 2051 2058 10.1128/AEM.02378-07 18281436 PMC2292594

[B33] Sonne-Hansen J Ahring BK Thermodesulfobacterium hveragerdense sp. nov., and Thermodesulfovibrio islandicus sp. nov., two thermophilic sulfate reducing bacteria isolated from a Icelandic hot spring Syst Appl Microbiol 1999 22 559 564 10.1016/S0723-2020(99)80009-5 10794144

[B34] Portillo M-C Cuecas A Pasomsup P Kanoksilapatham W Gonzalez J-M Distribution of baterial communities along a temperature gradient in Mae Fang Hot Springs (Thailand) GenBank Database 2010 http://www.ncbi.nlm.nih.gov/nuccore/HQ416834.1

[B35] Haouari O Fardeau ML Cayol JL Fauque G Casiot C Elbaz-Poulichet F Hamdi M Ollivier B Thermodesulfovibrio hydrogeniphilus sp. nov., a new thermophilic sulphate-reducing bacterium isolated from a Tunisian hot spring Syst Appl Microbiol 2008 31 38 42 10.1016/j.syapm.2007.12.002 18221850

[B36] Yan T Zhu L Mao Y Ye N Zhang X Fang J Diversity and distribution of bacterial community composition in Nansha Bay sediment in response to fish cage farming GenBank Database 2011 http://www.ncbi.nlm.nih.gov/nuccore/JQ217253.1

[B37] Liu R Li D Gao Y Zhang Y Wu S Ding R Hesham AEL Yang M Microbial diversity in the anaerobic tank of a full-scale produced water treatment plant Process Biochem 2010 45 744 751 10.1016/j.procbio.2010.01.010

[B38] Patel BKC Morgan HW Daniel RM Fervidobacterium nodosum gen. nov. and spec. nov., a new hemoorganotrophic, caldoactive, anaerobic bacterium Arch Microbiol 1985 141 63 69 10.1007/BF00446741

[B39] Andrews KT Patel BK Fervidobacterium gondwanense sp. nov., a new thermophilic anaerobic bacterium isolated from nonvolcanically heated geothermal waters of the Great Artesian Basin of Australia Int J Syst Bacteriol 1996 46 265 269 10.1099/00207713-46-1-265 8573506

[B40] He YL Ding YF Long YQ Two cellulolytic Clostridium species: Clostridium cellulosi sp. nov. and Clostridium cellulofermentans sp. nov Int J Syst Bacteriol 1991 41 306 309 10.1099/00207713-41-2-306 1854643

[B41] Weller R Bateson MM Heimbuch BK Kopczynski ED Ward DM Uncultivated cyanobacteria, Chloroflexus-like inhabitants, and spirochete-like inhabitants of a hot spring microbial mat Appl Environ Microbiol 1992 58 3964 3969 1282313 10.1128/aem.58.12.3964-3969.1992 PMC183212

[B42] Daumas S Cord-Ruwisch R Garcia JL Desulfotomaculum geothermicum sp. nov., a thermophilic, fatty acid-degrading, sulfate-reducing bacterium isolated with H2 from geothermal ground water Antonie Van Leeuwenhoek 1992 54 165 178 3395110 10.1007/BF00419203

[B43] Miroshnichenko ML Lebedinsky AV Chernyh NA Tourova TP Kolganova TV Spring S Bonch-Osmolovskaya EA Caldimicrobium rimae gen. nov., sp. nov., an extremely thermophilic, facultatively lithoautotrophic, anaerobic bacterium from the Uzon Caldera, Kamchatka Int J Syst Evol Microbiol 2009 59 1040 1044 10.1099/ijs.0.006072-0 19406789

[B44] Albuquerque L Franca L Rainey FA Schumann P Nobre MF Costa MS Gaiella occulta gen. nov., sp. nov., a novel representative of a deep branching phylogenetic lineage within the class Actinobacteria and proposal of Gaiellaceae fam. nov. and Gaiellales ord. nov Syst Appl Microbiol 2011 34 595 599 10.1016/j.syapm.2011.07.001 21899973

[B45] Lau MC Aitchison JC Pointing SB Bacterial community composition in thermophilic microbial mats from five hot springs in central Tibet Extremophiles 2009 13 139 149 10.1007/s00792-008-0205-3 19023516

[B46] Iino T Nakagawa T Mori K Harayama S Suzuki K Calditerrivibrio nitroreducens gen. nov., sp. nov., a thermophilic, nitrate-reducing bacterium isolated from a terrestrial hot spring in Japan Int J Syst Evol Microbiol 2008 58 1675 1679 10.1099/ijs.0.65714-0 18599715

[B47] Auchtung TA Shyndriayeva G Cavanaugh CM 16S rRNA phylogenetic analysis and quantification of Korarchaeota indigenous to the hot springs of Kamchatka, Russia Extremophiles 2011 15 105 116 10.1007/s00792-010-0340-5 21153671

[B48] Tomova I Dimitrova D Stoilova-Disheva M Lyutskanova D Kambourova M Archael diversity at two hot springs, Rupi Basin, Bulgaria Biotechnol Biotechnol Equip 2011 25 105 113 10.5504/BBEQ.2011.0120

[B49] Sahl J-W Gary M-O Harris J-K Spear J-R A comparative molecular analysis of phreatic limestone sinkholes in northeastern Mexico GenBank Database 2009 http://www.ncbi.nlm.nih.gov/nuccore/FJ901693.1

[B50] Huang Q Dong CZ Dong RM Jiang H Wang S Wang G Fang B Ding X Niu L Li X Zhang C Dong H Archaeal and bacterial diversity in hot springs on the Tibetan Plateau, China Extremophiles 2011 15 549 563 10.1007/s00792-011-0386-z 21695489

[B51] Pester M Schleper C Wagner M The Thaumarchaeota: an emerging view of their phylogeny and ecophysiology Curr Opin Microbiol 2011 14 300 306 10.1016/j.mib.2011.04.007 21546306 PMC3126993

[B52] Wemheuer B Taube R Akyol P Wemheuer F Daniel R Microbial diversity and biochemical potential encoded by thermal spring metagenomes derived from the kamchatka peninsula Archaea 2013 136714 23533327 10.1155/2013/136714 PMC3600328

[B53] Swingley WD Meyer-Dombard DR Shock EL Alsop EB Falenski HD Havig JR Raymond J Coordinating Environmental Genomics and Geochemistry Reveals Metabolic Transitions in a Hot Spring Ecosystem PLoS ONE 2012 7 e38108 10.1371/journal.pone.0038108 22675512 PMC3367023

[B54] Varfolomeev SD Molecules in extreme environments Taк и ocтaeтcя 2013 in press

[B55] Kolmogorov YP Trounova VA Analytical potential of EDXRF using toroidal focusing systems of highly oriented pyrolytic graphite (HOPG) X-ray Spectrom 2002 31 432 436 10.1002/xrs.603

[B56] Schmieder R Edwards R Quality control and preprocessing of metagenomic datasets Bioinformatics 2011 27 863 864 10.1093/bioinformatics/btr026 21278185 PMC3051327

[B57] Caporaso JG Kuczynski J Stombaugh J Bittinger K Bushman FD Costello EK Fierer N Pena AG Goodrich JK Gordon JI Huttley GA Kelley ST Knights D Koenig JE Ley RE Lozupone CA McDonald D Muegge BD Pirrung M Reeder J Sevinsky JR Turnbaugh PJ Walters WA Widmann J Yatsunenko T Zaneveld J Knight R QIIME allows analysis of high-throughput community sequencing data Nature methods 2010 7 335 336 10.1038/nmeth.f.303 20383131 PMC3156573

[B58] Edgar RC Search and clustering orders of magnitude faster than BLAST Bioinformatics 2010 26 2460 2461 10.1093/bioinformatics/btq461 20709691

[B59] Edgar RC Haas BJ Clemente JC Quince C Knight R UCHIME improves sensitivity and speed of chimera detection Bioinformatics 2011 27 2194 2200 10.1093/bioinformatics/btr381 21700674 PMC3150044

[B60] DeSantis TZ Hugenholtz P Larsen N Rojas M Brodie EL Keller K Huber T Dalevi D Hu P Andersen GL Greengenes, a chimera-checked 16S rRNA gene database and workbench compatible with ARB Applied and environmental microbiology 2006 72 5069 5072 10.1128/AEM.03006-05 16820507 PMC1489311

